# Letter from Paris

**Published:** 1868-06-01

**Authors:** 


### CHICAGO MEDICAL JOURNAL.

*Vol. XXV.--JUNE* 1, 1868.—*No.* 11.

### *FOREIGN CORRESPONDENCE.*

PARIS, March 27th, 1868.

THE Academy of Medicine, at its last sitting, elected Dr. Davaine as member in the place of the late Dr. Trousseau. Dr. Davaine is one of the attending physicians at the Palace of the Tuilleries. M. Reiset has published an article on the *Respiration of Cattle*, giving an account of certain experiments of his to ascertain the effect of change of food on the nature of the breath emitted. From these experiments it appears that calves feeding on good grass exhale more nitrogen, and a normal quantity of proto-carburetted hydrogen; the latter at the rate of four-thirds of a litre, and the former at the rate of six grammes and seven-tenths per hour. Calves, on the contrary, exclusively fed on milky substances, and deprived of vegetable food, exhale gaseous substances analogous to those exhaled by carnivora. The quantity of proto-carburetted hydrogen is, then, next to nothing, and M. Reiset is of opinion that this gas is produced only by vegetable food, when in a state of fermentation and elaboration in the first stomach of ruminants fed in the common way. On the other hand, in the case of an exclusively milky diet, the exhalation of nitrogen is also very considerable, and nearly double what it is under the influence of vegetable diet. On French farms it is the custom to feed calves that are to be fattened with milk or curd mixed up with a certain quantity of well boiled rice. This hydro-carburetted substance, added to the other food, affords respiration materials that are easily consumed, and thus moderate the destruction of plastic matter, rich in nitrogen. Hence the produce of respiration depends much more on the nature of the food administered, than on the species of animal. M. Reiset also makes some remarks on the disease called the *butts*, which often occurs when cattle have eaten too greedily of clover. Some two or three hundred head of cattle may thus, in the course of half an hour, be all at once exposed to certain death. In such cases, M. Reiset recommends the quick absorbtion of the carbonic acid, which is almost exclusively the cause of the affection, by means of calcined magnesia, or else saccharite of lime.

The question of the purity of the water we drink has long attracted the attention of hygienists, and not without reason, since it is now proved that the most fearful epidemics may have no other origin than that of putrescent organic matter suspended or dissolved in the water used for culinary purposes or drink. M. Bellamy devotes an article in the *Journal des Connoissances Medicales* to the important subject, and proposes the sub-sulphate alumina as a proper test for water. The organic particles contained in the liquid are, he informs us, of the same nature as what is generally termed *humus*, or the active matter of garden-mould. They are incapable of crystalization, are of very indefinite composition, more or less of a brown color, and capable of forming, with albumen, certain insoluble lakes, which, by their depth of hue, may serve to denote the quantity of organic matter contained in a given kind of water. Alum has been used as a test, but unless the liquid be very impure that salt is not readily decomposed, and the sub-sulphate of alumina is preferable. It may be prepared by adding 12 cubic centimètres of a solution of caustic potash, of the strength of 10 per cent., to a solution of 8 grammes of alum in 100 of water. A precipitate is thus formed, which is slowly re-dissolved, and the solution will keep indefinitely in a limpid state. This subsulphate contains about half as much more potash than alum, which is a double salt. Of this solution, four cubic centimètres are poured into a litre of the water, to be tested. The decomposition of the salt takes place under the triple influence of the mass of water, the earthy bicarbonates, and organic matter contained in it. The latter falls to the bottom of the vessel in the course of a few hours, being precipitated by the alumina, which combines with it. It is true that all the organic matter contained in the water will not be precipitated in this way, on account of the many elements of which it is composed, and a few of which may remain in suspension; but those derived from sewers will chiefly be fastened upon by the alumina, and it is to these the colored particles mainly belong. It must be kept in mind that this prepared method is not intended for a quantitative analysis, but merely as an easy way of detecting the foulness of water in a very short time.

M. Becquerel has published a paper on electro-capillary phenomena. Having previously shown that when a slip of filtering paper is placed between two glass plates, in order to effect the slow efflux of a metalic solution contained in a vessel dipping into another solution, the electro-capillary action is rendered more easy. The author illustrates this fact by a variety of experiments. Thus, if moist persulphuret of iron be deposited on a copper lamina placed between two glass ones, and if the borders be covered with putty to prevent the entrance of atmospheric air, the sulphuret of iron will be gradually decomposed, sulphuret of copper will be formed, and iron in a metallic state will be deposited here and there, and this effect is attributable to electro-capillary action.

M. Schlœsing has communicated to the Academy of Science the result of his researches on the origin of the nitrous gas which is evolved during the fermentation of the juice of the beet root. This nitrous gas he shows to be due to the reduction of the nitrates contained in vegetable juices. One of his predecessors at the laboratory of the Tobacco Manufactory, M. Rey had proved that tobacco juice left to putrefy in a close vessel, evolved protoxide of nitrogen; this gas was diluted with carbonic acid, and its proportion varied according to the quality of the tobacco. M. Schlœsing, acting upon the hint, has instituted experiments, from which it appears that the nitrates contained in the juice are decomposed during putrefaction, and rapidly enough to allow of daily analysis of the produce. In a vegetable juice containing nitrates, the gas evolved leaves behind, after the absorption of its carbonic acid, a residue inclosing protoxide of nitrogen, a substance which will not be found in the residue unless the juice contained nitrates, and, agreeably to this, during the putrefaction of tobacco juice, there is a connexion between the destruction of the nitrates and the generation of the protoxide, nor does one of the phenomena take place without the other. One author has extended his experiments to other substances. Thus, sugar and water, during the lactic fermentation, will evolve nothing but carbonic acid and hydrogen; but if nitrate of potash be put in, the result will be a mixture of carbonic acid, nitrogen and protoxide and binoxide of nitrogen. Fresh leaves and roots, left exposed to the open air, in dilute solutions of some nitrate, were found to decompose nitric acid when the smell of liquids betrayed the commencement of putrefaction.

In an essay on the sub-nitrate of bismuth, Dr. Monneret enumerates the various effects of this valuable medicine. He affirms he was the first to employ it in nose-bleeding and intestinal hæmmorrhage, in which latter case he administers a teaspoonful of it in two table-spoonfuls of water once an hour. He has used it in typhus fever for the last five years, and never, during that time, has he lost a single patient by intestinal hæmmorrhage. The same salt appears to be a specific for the cure of ozæna and otorrhœa. Sub-nitrate of bismuth, in his opinion, only acts negatively, and merely as an insulating agent, but it prepares the mucous membrane for the prompt absorbtion of remedies, the action of which is uncertain.

The *Moniteur* relates a case of poisoning by mushrooms, from which it would appear that this dangerous cryptogamous plant is capable of causing temporary madness. This was observed a few days ago in the case of two young men who had been gathering mushrooms in the forest of Marchiennes, and who cooked them without taking any of the ordinary precautions. The consequence was that they were seized with a violent attack of madness, and were with difficulty brought home to their family, where they recovered their senses after proper medical treatment.

Dr. Piorry, who has been seriously ill for some time past, is now quite recovered, and has resumed his clinical lectures.

